# Critical Care Aggression and Violence Events (CAVE) Against Nurses in the ICU: Epidemiology and Risk Factors for Recurrence

**DOI:** 10.1097/CCM.0000000000007264

**Published:** 2026-07-24

**Authors:** Anja I. Frei, Sebastian Berger, Pascale Grzonka, Simon A. Amacher, Paulina Kliem, Caroline E. Gebhard, Sabina Hunziker, Raoul Sutter

**Affiliations:** 1 Clinic for Intensive Care, Department of Acute Medicine, University Hospital Basel, Basel, Switzerland.; 2 Interdepartmental Division of Critical Care, University Health Network, Toronto, ON, Canada.; 3 Department of Anesthesiology and Intensive Care Medicine, Medical Centre—University of Freiburg, Faculty of Medicine, University of Freiburg, Freiburg im Breisgau, Germany.; 4 Medical Faculty, University of Basel, Basel, Switzerland.; 5 Institute for Intensive Care Medicine, Stadtspital Zürich, Zürich, Switzerland.; 6 Medical Communication and Psychosomatic Medicine, University Hospital Basel, Basel, Switzerland.; 7 Department of Clinical Research, University of Basel, Basel, Switzerland.

**Keywords:** aggression, critical care nursing, intensive care units, recurrence, workplace violence

## Abstract

**OBJECTIVES:**

To quantify and characterize aggression and violence against nurses, identify associated clinical characteristics, evaluate measures in response, and explore predictors of recurrent incidents in the ICU.

**DESIGN:**

Retrospective single-center study. Incidence and characteristics of aggression and violence against ICU nurses were the primary outcomes. Aggression was defined as hostile behavior or resistance to care without intent to harm. Violence comprised verbal, physical, or sexual acts to threaten or inflict harm. Secondary outcomes included risk factors for recurrent incidents, defined as multiple episodes of aggression/violence separated by greater than or equal to 24 hours. Multivariable analyses were performed to identify potential independent associations.

**SETTING:**

Data were collected from 2023 to 2024 at the tertiary medical-surgical ICU with up to 35 beds at the University Hospital Basel. Nursing operations followed a three-shift system: day (07:00–15:00), late (15:00–23:00), and night (23:30–07:00).

**SUBJECTS:**

Clinical reports from ICU nurses were screened.

**INTERVENTIONS:**

None.

**MEASUREMENTS AND MAIN RESULTS:**

During 2190 shifts, 308 aggressive or violence incidents (14% of shifts) were reported. Most occurred during late (37%) or night (34%) shifts; 75% involved violence. Violent incidents were combined in nature (79% physical, 43% verbal, and 3% sexual). Patients were mainly involved (97%) and predominantly male (71%; median age 67; 90% emergency admissions). Psychiatric comorbidities, drug, and alcohol abuse were found in 20–33%. Recurrent aggression/violence occurred in 27%, predicted by longer ICU stay (adjusted odds ratio [OR] = 1.05/every additional hour), aggression as the first incident (adjusted OR = 6.9), and verbal violence as the first incident (adjusted OR = 4.3). Nurses (89%, two-thirds female, median 7 yr’ experience) rarely documented harm (1.3%), but patients were reported to suffer complications in 60% of incidents, mainly from oversedation and physical harm from restraints.

**CONCLUSIONS:**

Violence in the ICU is frequent and almost always caused by patients. Recurring violence was independently associated with longer ICU stay, initial aggression, and verbal violence. Staff harm was rare, but incidents were frequently associated with complications in patients.

**KEY POINTS**

**Question**: What are the incidence and characteristics of aggression and violence against ICU nurses, and what factors are associated with recurrent incidents?

**Findings**: In this retrospective cohort study, 308 incidents occurred during 14% of ICU shifts, most during late or night shifts, and 75% involved violence. Patients were involved in 97% of cases, and recurrent incidents occurred in 27%. Longer ICU stay, initial aggression, and initial verbal violence were statistically significantly associated with recurrence.

**Meaning**: Aggression and violence in the ICU are frequent, predominantly patient-initiated, and early incident characteristics may help identify patients at risk for recurrence.

Healthcare workers face a higher risk of workplace violence (WPV) compared with other occupational groups. The international organizations and opinion leaders defined WPV in nursing as any verbal, physical, or sexual act, incident, or behavior that departs from reasonable conduct in which a person is assaulted, threatened, harmed, or injured in the course of, or as a direct result of, their work (1–3).

WPV is associated with poor caregivers’ health, longer absence of work and a high turnover intention, exacerbating ICU professionals’ shortage (4–7), and reduced quality of patient care (8, 9).

While emergency and psychiatric departments have been the focus of research on WPV, the ICU setting remains understudied despite presenting unique challenges that increase vulnerability to violence (9), such as physical restraints and invasive procedures. Families and patients are exposed to emotional strain, conflicting views regarding treatment decisions and prognosis and pressure of complex decision-making (10, 11).

Despite increasing awareness, evidence is limited by mainly qualitative studies, whereas quantitative analyses with reliable statistics on frequency, recurrence, and risk factors are scarce. This limits the ability to develop prevention strategies and risk assessments (11, 12).

We aimed to quantify and characterize the nature of aggression and/or WPV against ICU nurses and to identify clinical characteristics of both subjects involved. We further sought to evaluate the interventions and response measures and to explore potential predictors for recurrent aggression and/or WPV after initial episodes.

## METHODS

### Design, Ethics, and Approvals

This retrospective cohort study was performed at the ICU for adult patients of the University Hospital Basel, a Swiss academic tertiary medical-surgical ICU with up to 35 beds. During the study, ICU nursing staff included 296 nurses (214 [72.3%] specialized ICU nurses and 82 [27.7%] registered nurses) within a three-shift system comprising a duration of 8 hours per shift (i.e., day [07:00–15:00], late [15:00–23:00], and night [23:00–07:00]). During the study, our unit employed a standardized nurse-to-patient model with 23 nurses on day shifts, 22 on late shifts, and 21 on night shifts. This staffing supported up to 20 patients defined as “level 3 patients” requiring invasive monitoring, mechanical devices, or vasopressors. All registered nurses were enrolled in closely supervised postgraduate programs to obtain final ICU specialization.

The local ethics committee (Institutional Review Board [IRB] waiver number BASEC_ID Req-2024-00927) waived patients’ consent and no IRB approval was necessary. The protocol was registered (NCT06921083). The study reporting followed the Strengthening the Reporting of Observational Studies in Epidemiology guidelines (13).

### Definitions and Assessment

Aggression was defined as nondirected hostile or agitated behavior (e.g., rude remarks, resistance to care) lacking intent to harm.

WPV was defined, as recommended by international organizations and opinion leaders as actions, or behavior departing from reasonable conduct in which a healthcare worker is assaulted, threatened, harmed, or injured during or as a result of their work (1–3). Consistent with prior studies, violence was categorized as verbal, nonverbal, physical, and sexual, including attempted and completed acts (9, 14–16). Verbal violence involved abusive, insulting, or threatening expressions, including shouting, slurs, and threats of harm. Nonverbal violence consisted of behaviors conveying harm without speech, such as menacing postures or throwing objects toward staff. Physical violence was defined as actual or attempted physical contact (e.g., hitting, biting, scratching, spitting), or the use of objects/fluids likely to cause injury or fear. Sexual violence encompassed unwanted behavior with sexual content, including verbal harassment, nonconsensual touching, and indecent exposure. Violence following aggression was used for patients who exhibited aggressive behavior before WPV.

Recurrent WPV was defined as multiple episodes within an ICU stay separated by at least 24 hours of WPV-free intervals. Patient’s clinical status was quantified every 8 hours using the Richmond Agitation-Sedation Scale (RASS) and the Intensive Care Delirium Screening Checklist (ICDSC).

### Reporting and Data Collection

During the study, nurses had the opportunity to document incidents of aggression and/or WPV for every shift using the password-protected electronic data system MetaVision (Version 5.46.44, iMDsoft, Wakefield, MA) Each shift-end reports mandated structured free-text documentation of medical, social, and ethical aspects. The neurology/psychology section recommended standardized entries for patient’s clinical status, cooperation, motor activity, mood, and communication, alongside reporting of restrictive measures and their indications (i.e., aggression, violent behavior). Categorization of events relied on the clinical discretion of primary caregivers. To preserve the authenticity of spontaneous documentation and avoid potential reporting bias no additional study-specific reporting guidance was provided.

From January 2023 to December 2024, all shift reports were screened for aggression and/or WPV, using a custom R script (Version 4.4.0 [2024-04-24], RStudio: Boston, MA) based on three predefined criteria. Cases were identified if they matched greater than or equal to 1 of the following (**Fig. 1**): 1) keywords within reports related to aggression or WPV (**Supplemental Table 1**, https://links.lww.com/CCM/I20); 2) reports from shifts in which the patients’ had RASS greater than or equal to 2 (indicating being agitated [=2], very agitated [=3], or combative [=4]); or 3) ICDSC greater than or equal to 1 (consisting of agitation, inappropriate speech, or hallucinations). Flagged reports were reviewed by (A.I.F.) and forwarded to (S.B.) for confirmation. Discrepancies were resolved through consensus or consultation with the senior investigator (R.S.).

**Figure 1.**
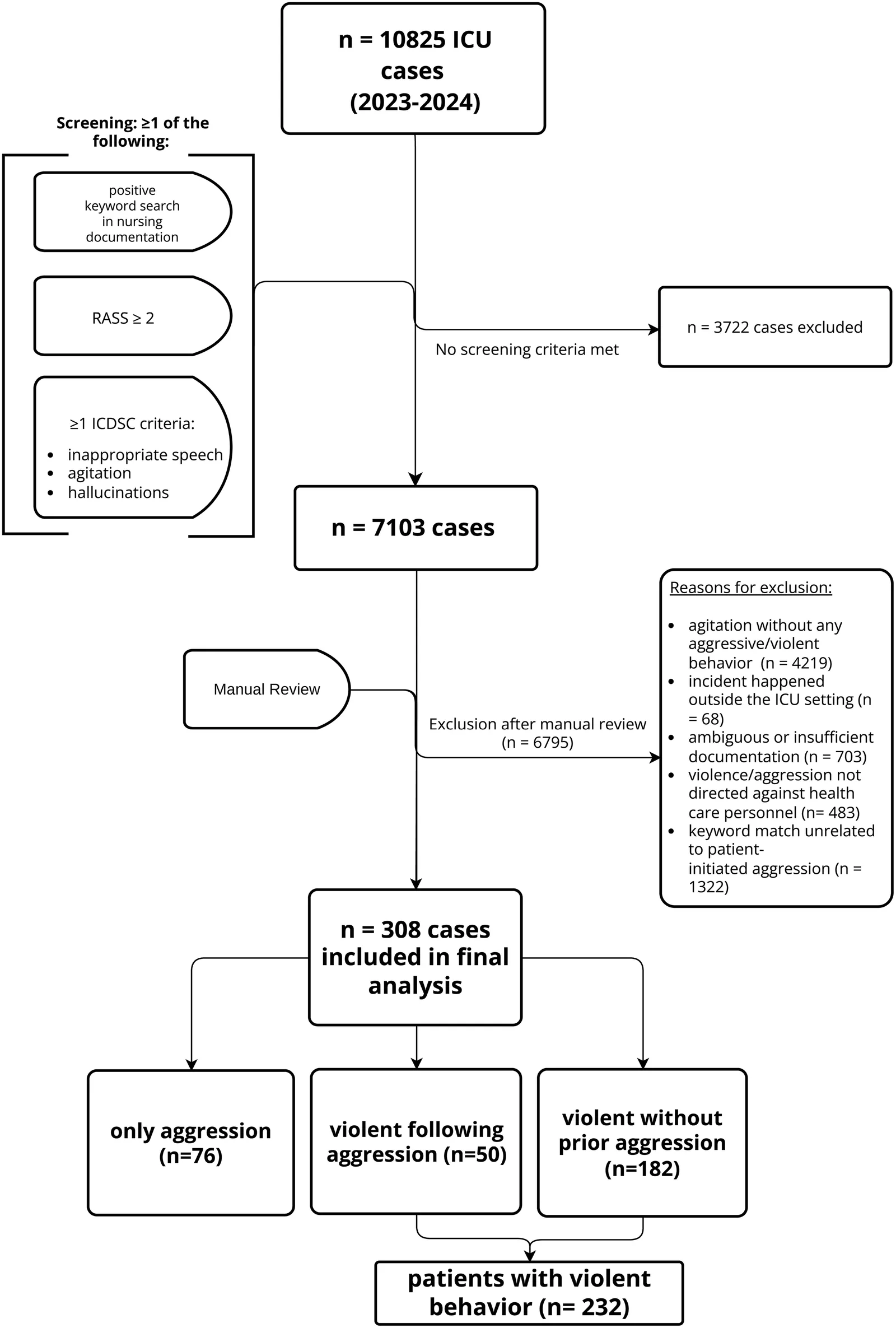
Flowchart. ICDSC = Intensive Care Delirium Screening Checklist, RASS = Richmond Agitation-Sedation Scale.

In cases of reported aggression and/or violence, all documentation related to these incidents and the patient-specific medical records of current and past in-hospital treatment were manually reviewed to further assess prior aggression and/or WPV. During the study period and as routine clinical practice all invasive measures in response to such incidents, including sedation and physical restraints, were governed by a standardized multiprofessional protocol and were never initiated by nurses autonomously. This MetaVision-based protocol required a multiprofessional assessment and order from the treating physician. Consequences for patients and staff, including measures and treatment modifications, were retrieved.

### Endpoints

The primary objective was to quantify and characterize the nature of aggression and/or WPV reported against ICU nurses. The secondary objectives were to identify clinical characteristics of involved individuals (patients and/or relatives) and nurse victims, to describe interventions and measures, and to determine associations with aggression or WPV (as a fixed outcome).

### Statistics

Continuous variables are reported as medians with interquartile ranges (IQRs); categorical variables are reported as counts and percentages.

Univariable comparisons were performed to assess differences regarding characteristics between cases with single and multiple acts of aggression and/or violence. Comparisons of categorical variables were conducted using Pearson chi-square test or Fisher exact test. Continuous variables were compared using the Mann-Whitney *U* test. To account for multiple testing/comparisons (*n* = 60), the Bonferroni correction was applied (17, 18), setting significance at a two-sided *p* value of less than or equal to 0.001.

Multivariable logistic regression was conducted to identify independent associations with repeated aggressive behavior and/or WPV. Variables with significant differences in univariable analyses were included in the models. Model calibration was assessed using the Hosmer-Lemeshow chi-square goodness-of-fit test (19).

Analyses were performed using Stata, Version 16.1 (StataCorp LLC, College Station, TX).

## RESULTS

Characteristics of aggression and/or WPV are presented in **Table 1**. Overall, 308 reports were identified (14% of 2190 shifts). Among these, the median time from ICU admission to the first episode was 1.6 days. Most occurred during late (37%) or night shifts (34%).

**TABLE 1.** Characteristics of All Incidents of Aggression and/or Violence in the ICU (*n* = 308)

Clinical Variables Around Incidents of Aggression and/or Violence	*n* (%) or Median (IQR)
Time to incident from admission, d, median (IQR)	1.55 (0.5–4)
Aggressive behavior without violence, *n* (%)	76 (24.7)
Aggression before violence, *n* (%) [% of 232]	50 (16.2) [21.6]
Primary violent events, *n* (%)	232 (75.3)
Physical, *n* (%) [% of 232]	181 (58.8) [78]
Verbal, *n* (%) [% of 232]	99 (32.1) [42.7]
Sexual, *n* (%) [% of 232]	8 (2.6) [3.4]
Nonverbal, *n* (%) [% of 232]	7 (2.3) [3.0]
Type of shift during incident	
Day	89 (28.9)
Late	115 (37.3)
Night	104 (33.8)
Time since last shift, hr, median (IQR)	3 (1.4–5.1)
Glasgow Coma Score (ranging from 3 to 15) at incident, median (IQR)	13 (12–14)
RASS at incident, median (IQR)	4 (2–4)
Highest RASS day of incident, median (IQR)	4 (3–4)
Highest RASS day before incident, median (IQR)	1 (0–3)
Highest RASS day after incident, median (IQR)	2 (1–3)
ICDSC at incident, median (IQR)	5 (4–7)
ICDSC day before incident, median (IQR)	5 (3–6)
ICDSC day after incident, median (IQR)	6 (4–7)
Documented presumed reason for incident, *n* (%)	74 (24)
Visit from family, *n* (%) [% of 74]	16 (5.2) [21.6]
Received phone call, *n* (%) [% of 74]	7 (2.3) [9.5]
Received bad news, *n* (%) [% of 74]	0 (0.0) [0.0]
Other, *n* (%) [% of 74]	54 (17.5) [72.9]
Restrained at incident, *n* (%)	91 (29.6)
Additional violent incidents after first violent event, *n* (% of 232)	43 (18.5)
Physical, *n* (% of 43)	35 (81.4)
Verbal, *n* (% of 43)	24 (55.8)
Sexual, *n* (% of 43)	4 (9.3)
Nonverbal, *n* (% of 43)	2 (4.7)
Number of additional incident, mean (sd)	0.3 (0.8)

ICDSC = Intensive Care Delirium Screening Checklist, IQR = interquartile range, RASS = Richmond Agitation-Sedation Scale.

Most incidents (75%) involved violence, primarily of physical (79% of violent incidents) or verbal nature (43%), whereas sexual (3%) and nonverbal (2%) acts were rare. Aggression without violence accounted for 25% of incidents and in 16% aggression emerged before WPV. Presumed triggers were identified in one quarter of events, mostly family visits. About one third of patients were restrained during incidents.

### Characteristics of Involved Individuals and Victims

Involved individuals exhibiting aggression and/or WPV were almost exclusively patients (97%), with few involving relatives. Patients had a median age of 67 years, were predominantly male (71%), and most were emergency admissions (90%; **Supplemental Table 2**, https://links.lww.com/CCM/I20). Roughly one in six patients had aggression or WPV before ICU admission, and nearly one in five WPV incidents recurred during the same admission, mainly as repeated physical assaults. During incidents, patients were generally awake and agitated (median RASS +4, ICDSC 5; Table 1). Neurologic diseases were most common (29%), followed by cardiovascular (18%) and infectious diseases (12%). Nearly one-third had psychiatric comorbidities, one-third had alcohol abuse, and one0fifth had drug use.

Victims were mainly experienced nurses (89%; median 7-yr experience) and two-thirds being female (**Supplemental Table 3**, https://links.lww.com/CCM/I20). Roughly, 14% of victims experienced repeated incidents by the same patient.

### Measures and Complications

Interventions subsequent to incidents were implemented in all cases (**Table 2**). Verbal soothing (64%) and pharmacological strategies, particularly sedatives (62%) and antipsychotics (43%), were most frequent. Restraints were used in over one third, and one-to-one nursing was required in nearly one quarter. Security staff were rarely called (6%).

**TABLE 2.** Characteristics of Measures and Complications Following Aggression and/or Violence in the ICU (*n* = 308)

Characteristics of Measures and Complications Following Incidents of Aggression and/or Violence	*n* (%)
Measures taken	308 (100)
Called other nurses	23 (7.5)
Called doctor	36 (11.7)
Called security	17 (5.5)
Called police	0 (0.0)
Verbal soothing	198 (64.3)
Involved family	30 (9.7)
Administered sedatives	191 (62.0)
Administered antipsychotics	132 (42.9)
Applied restraints	112 (36.4)
Offered distraction (television, music)	19 (6.2)
One on one nursing	70 (22.7)
Other	27 (8.8)
Sequelae for victims documented	
Physical injury	4 (1.3)
Emotional injury	0 (0.0)
Leave from work	0 (0.0)
Complications for patient	
More tubes/lines/catheters	4 (1.3)
Physical injuries	3 (1.0)
Emotional injuries	6 (2.0)
Aspiration	1 (0.3)
Pain	2 (0.7)
Agitation	12 (3.9)
Restraint	40 (13.0)
Other	13 (4.2)

For nurses, sequelae were rarely documented (Table 2) with 1.3% physical injuries. Patient complications were common including aspiration, increasing agitation, physical/emotional injury, aspiration, or pain.

### Potential Factors for Recurrent Events

Patients exhibiting multiple acts of aggression and/or WPV (27%) differed from those with single events (**Tables 3**, **4** and **5**). They had longer ICU stays, more often displayed aggression without WPV at initial episodes, and were more often male. Their violent behavior tended to be more severe, with higher combined proportions of physical, verbal, and sexual assaults. They were restrained and treated with sedatives and antipsychotics more often.

**TABLE 3.** Univariable Comparisons of Demographics and Clinical Characteristics of Involved Individuals With Aggressive and/or Violent Behavior Between Case of Singular and Repetitive Incidents (*n* = 308)

Characteristics	Single Incident of Aggression/Violence (*n* = 225)	Multiple Incidents of Aggression/Violence (*n* = 83)	*p* ^a^
Involved individuals			
Age, yr, median (IQR)	68 (52–78)	63 (45–75)	0.09^b^
Male sex, *n* (%)	153 (68)	67 (80.7)	0.028^c^
ICU length of stay, d, median (IQR)	3.5 (1.6–8.0)	7.4 (3.7–15.8)	**< 0.001** ^b^
Prior ICU stays, *n* (%)	93 (41.3)	30 (36.1)	0.41^c^
Aggressive behavior, *n* (%)	76 (33.8)	50 (60.2)	**< 0.001** ^b^
Type of initial violence, *n* (%)			
Physical	121 (53.8)	60 (72.3)	0.003^c^
Verbal	59 (26.2)	40 (48.2)	**< 0.001** ^c^
Sexual	2 (0.9)	6 (7.2)	0.006^d^
Nonverbal	0 (0.0)	7 (8.4)	**< 0.001** ^d^
Postoperative admission, *n* (%)	59 (26.2)	19 (22.9)	0.55^c^
Emergency admission, *n* (%)	196 (87.1)	80 (96.4)	0.019^d^
Swiss nationality, *n* (%)	172 (76.4)	54 (65.0)	0.045^c^
Main diagnosis, *n* (%)			0.29^d^
Neurologic disease	62 (27.6)	26 (31.3)	
Psychiatric disease	13 (5.8)	6 (7.2)	
Metabolic disease	32 (14.2)	8 (9.6)	
Infectious disease	32 (14.2)	5 (6.0)	
Cardiovascular disease	40 (17.8)	15 (18.1)	
Gastrointestinal disease	10 (4.4)	4 (4.8)	
Pulmonary disease	20 (8.9)	9 (10.8)	
Trauma	9 (4.0)	8 (9.6)	
Charlson Comorbidity Index, median (IQR)	4 (2–6)	4 (1–6)	0.99^b^
Simplified Acute Physiology Score 2 (ranging from 0 to 163), median (IQR)	35 (27–45)	36 (28–43)	0.55^b^
Glasgow Coma Score (ranging from 3 to 15) at incident, median (IQR)	13 (12–14)	13 (12–14)	0.90^b^
Richmond Agitation-Sedation Scale at incident, median (IQR)	4 (2–4)	4 (3–4)	0.16^b^
Intensive Care Delirium Screening Checklist at incident, median (IQR)	5 (3–6)	6 (5–7)	0.030^b^
Positive toxicologic screening, *n* (%)	30 (13.3)	13 (15.7)	0.60^c^
History of neurologic disease, *n* (%)	76 (33.8)	33 (39.8)	0.33^c^
History of psychiatric disease, *n* (%)	63 (28)	25 (30.1)	0.72^c^
History of alcohol abuse, *n* (%)	71 (31.6)	35 (42.2)	0.08^c^
History of drug abuse, *n* (%)	43 (19.4)	18 (21.7)	0.62^c^
History of dementia, *n* (%)	26 (11.6)	9 (10.8)	0.86^c^
Violence before ICU stay, *n* (%)	39 (17.3)	13 (15.7)	0.73^c^

IQR = interquartile range.

a Level of significance adjusted using the Bonferroni correction for multiple comparisons and set at a significance level at a two-sided *p* ≤ 0.001 due to the Bonferroni correction.

b Mann-Whitney *U* test.

c Pearson χ^2^ test.

d Fisher exact test.

Boldface indicates statistical significance.

**TABLE 4.** Univariable Comparisons of Victims (i.e., ICU Healthcare Professionals) and Clinical Characteristics Between Case With Singular and Repetitive Incidents (*n* = 308)

Victim Characteristics	Single Incident of Aggression/Violence (*n* = 225)	Multiple Incidents of Aggression/Violence (*n* = 83)	*p* ^a^
ICU nurse, *n* (%)	199 (88.4)	74 (89.2)	0.86^c^
Registered nurse, *n* (%)	26 (11.6)	9 (10.8)	0.86^c^
Female sex, *n* (%)	151 (67.1)	53 (63.8)	0.59^c^
Age, yr, median (IQR)	36 (29–44)	38 (29–49)	0.52^b^
Working percentage, median (IQR)	90 (70–100)	90 (80–100)	0.96^b^
Working experience, yr, median (IQR)	7.2 (2.9–10.8)	7.7 (3.6–11.9)	0.22^b^
Swiss nationality, *n* (%)	69 (30.7)	30 (36.1)	0.36^c^
Clinical characteristics			
Time from admission to incident, d, median (IQR)	1.4 (0.3–3.8)	1.8 (1.0–4.9)	0.006^b^
Type of shift during incident, *n* (%)			0.69^c^
Day	62 (27.6)	27 (32.5)	
Late	86 (38.2)	29 (34.9)	
Night	77 (34.2)	27 (32.5)	
Time since last shift, hr, median (IQR)	3 (1.5–5.2)	3 (1.1–5)	0.96^b^
Restrained at first incident, *n* (%)	55 (24.4)	36 (43.4)	**0.001** ^c^
Measures taken, *n* (%)			
Called other nurses	11 (4.9)	12 (14.5)	0.005^c^
Called doctor	24 (10.7)	12 (14.5)	0.36^c^
Called security	13 (5.9)	4 (4.8)	1.00^d^
Verbal soothing	142 (63.0)	56 (67.5)	0.48^c^
Patient’s family involved	14 (6.2)	16 (19.3)	**0.001** ^c^
Administered sedatives	126 (56.0)	65 (78.3)	**< 0.001** ^c^
Administered antipsychotics	88 (39.1)	44 (53.0)	0.030^c^
Applied restraints	70 (31.1)	42 (50.6)	0.002^c^
Offered distraction (television, music)	10 (4.4)	9 (10.8)	0.04^c^
One on one nursing	47 (20.9)	23 (27.7)	0.20^c^
Patient’s complications, *n* (%)			
Escalation of sedation	115 (51.8)	57 (68.7)	0.006
More tubes/lines/catheters	2 (0.8)	2 (2.4)	0.29^d^
Less tubes/lines/catheters	8 (3.5)	9 (10.8)	0.010^c^
Physical injuries	2 (0.8)	1 (1.2)	1.00^d^
Emotional injuries	3 (1.3)	3 (3.6)	0.35^d^
Aspiration	0 (0.0)	1 (1.2)	0.27
Pain	0 (0.0)	2 (2.4)	0.07^d^
Agitation	8 (3.5)	4 (4.8)	0.74^4^

IQR = interquartile range.

a Level of significance adjusted using the Bonferroni correction for multiple comparisons and set at a significance level at a two-sided *p* ≤ 0.001 due to the Bonferroni correction.

b Mann-Whitney *U* test.

c Pearson χ^2^ test.

d Fisher exact test.

Boldface indicates statistical significance.

**TABLE 5.** Univariable and Multivariable Analyses Regarding Characteristics Associated With ICU Patients Being Repetitively Aggressive and/or Violent (*n* = 308)

Characteristics Associated With Patients’ Repetitively Aggressive and/or Violent	Univariable Analyses		Multivariable Analyses	
	Unadjusted OR (95% CI)	*p*	Adjusted OR (95% CI)	*p* ^a^
ICU length of stay (per every additional hour)	1.03 (1.01–1.06)	0.009	1.05 (1.02–1.07)	**< 0.001**
Aggressive behavior at first incident	2.97 (1.77–4.99)	**< 0.001**	6.89 (3.49–13.61)	**< 0.001**
Verbal violence at first incident	2.62 (1.55–4.42)	**< 0.001**	4.27 (2.18–8.37)	**< 0.001**
Restrained at first incident	2.37 (1.39–4.02)	**0.001**	2.09 (1.11–3.95)	0.023
Patient’s family involved following first incident	3.60 (1.67–7.76)	**0.001**	3.92 (1.53–10.01)	0.004
Sedatives administered following first incident	2.84 (1.58–5.09)	**< 0.001**	3.45 (1.70–7.01)	**0.001**

OR = odds ratio.

a Level of significance adjusted using the Bonferroni correction for multiple comparisons and set at a significance level at a two-sided *p* ≤ 0.001 due to the Bonferroni correction.

The Hosmer-Lemeshow χ^2^ goodness-of-fit test indicated adequate model fit (χ^2^ = 7.79; *p* = 0.455).

Boldface indicates statistical significance.

Victim characteristics were not associated with recurrent incidents.

In multivariable models (Table 5), longer ICU stay, initial aggression, and verbal violence during first incidents remained associated with recurrence independent of potential confounders.

### From Aggression to Violence

**Supplemental Tables 4** and **5** (https://links.lww.com/CCM/I20) present analyses regarding repetitive aggression and/or WPV. Of 126 patients initially being aggressive, 40% escalated to violence (**Supplemental Table 4**, https://links.lww.com/CCM/I20). Compared with those remaining nonviolent, these patients had higher median RASS (RASS +4 vs. +3) and ICDSC (6 vs. 4.5) scores. They were more often restrained, sedated, and developed this behavior later. Multivariable analysis did not identify any independent associations (**Supplemental Table 5**, https://links.lww.com/CCM/I20).

## DISCUSSION

In this study, 2190 shifts were screened, revealing 308 aggression or violence incidents (14%), most in late (37%) and night shifts (34%). Patients were involved in 97% of incidents. Seventy-five percent of incidents were violent. Sixteen percent followed prior aggression within the same admission despite measures taken. While our screening process may have excluded cases with inadequate documentation and relied on caregivers’ free-text introducing potential selection bias, this attrition likely results in an underestimation of the true incidence of aggression and violence. Consequently, our findings should be interpreted as a conservative representation of the behavioral challenges within the ICU setting. Underreporting is an issue with a pooled estimate of ~47% (11). An American study compared self-reports with administrative data of 2010 employees, revealing underreported aggression because nurses considered it “part of the job” or doubted reports would lead to change (20). Inadequate documentation on our study; however, led to exclusion in only 6% (703 of a total of 10,825 records; Figure 1). Since shift-end nursing reporting was mandatory and an explanation is a legal requirement for initiating management responses in Switzerland, such as the use of restraints or emergency sedation, the most severe and resource-intensive incidents are likely well captured. Safety concerns for colleagues in subsequent shifts are a strong incentive for stringent documentation, further counteracting underreporting. Our study involved high-acuity patients: 90% were emergency admissions, often exhibiting severe agitation and delirium. Key diagnoses were neurologic (29%) and cardiovascular (18%), with comorbidities including psychiatric disorders (32%), alcohol abuse (33%), and drug use (18%). Incidents peaked during late (37%) and night shifts (34%) likely due to “sundowning” and slightly leaner staffing. Our care team consisted mostly of highly experienced nurses (median 7-yr experience), potentially lowering reported violence rates and limiting generalizability to centers with different settings.

Recurrent WPV occurred in 27% of patients, who had longer ICU stays, were more often male, and showed more severe behaviors. Multivariable analysis identified independent care- and patient-related associations with recurrence (longer ICU stay, aggression before incident, and verbal violence at the first incident). Importantly, ICU length of stay also reflects time at risk for recurrent events, and the observed association should therefore be interpreted with caution, as it may partly represent increased opportunity for recurrence rather than a causal relationship. Effects of ICU stay appear modest but in the context of high-acuity care, incremental increases across large cohorts have implications for resource allocation, bed availability, and costs.

This detailed dataset adds new insights, particularly regarding potential predictors of recurrence of aggressive and/or violent behavior against ICU nurses, which, to the best of the authors’ knowledge, have not been tested in prior studies so far. Thereby, our research heightens professional awareness and provides a foundation for future warning/prediction systems and targeted prevention strategies that are currently scarce (12).

When compared with our systematic review and meta-analysis (11), covering 75 studies and 139,533 healthcare workers worldwide. WPV prevalence rates were higher and identified with pooled frequencies of 31% for physical violence, 57% for verbal violence, and 12% for sexual violence. In single studies, up to 97% of staff reported verbal abuse, and 82% reported assaults (11). This supposed discrepancy with our Basel cohort’s prevalence is likely due to methodological factors and differences of the ICU setting. Most previous studies were cross-sectional surveys with long recall windows and high risk of recall and selection bias. Furthermore, our staff was mostly experienced (median 7-yr experience) and had a better nurse-patient ratio, especially during late and night shifts, as in most studies reporting these characteristics so analyses confirming younger age (14, 21–30) and work experience (31–33), as identified in several studies was not possible.

Younger age, being a nurse, and having less work experience are identified as consistent correlations of WPV exposure across multiple studies with the latter being another explanation for the lower yet still high frequency of aggression and violence in our study. High staff experience, as in our cohort, has likely increased underreporting “normalized” events. This aligns with a Chinese study where nurses with younger than 1 year of experience had two- to nine-fold higher odds of reporting WPV than senior peers (34), and an American study showing a 1.4 relative risk for those with younger than 5 years’ experience vs. older than 15 years (35). Additionally, Arnetz et al (20) linked younger than 5 years of experience to a 0.42 odds ratio of underreporting in hospitals.

On the patient side, findings are more consistent. Across studies, male sex, older age, delirium, agitation, and substance involvement have been associated with WPV (11). A European multicenter study found delirium and agitation implicated in up to 80% of WPV (36) mirroring our finding that patients had high median RASSs and ICDSCs, and those escalating from aggression to violence had higher agitation and delirium scores than those who did not. However, multivariable analysis identified no associations for transformation.

In our study more incidents were reported from late and night shifts, aligning with cross-sectional data from Nepal and China showing up to two-fold increased odds of violence during night shifts in ICUs (33, 34, 37). Reviews of ICU delirium describe diurnal fluctuation with evening/night worsening (“sundowning”) that can precipitate aggression if unmet needs (pain, dyspnea, withdrawal) are not rapidly addressed (38). Disturbed circadian biology, suppressed/phase-shifted melatonin and altered cortisol rhythms, correlates with delirium, further predisposing to nighttime agitation (39). Operational factors at night amplify this vulnerability. Night shifts commonly run with leaner staffing, fewer senior clinicians immediately available and slower access to support, all of which can delay de-escalation. Studies links overtime and night scheduling with more violence, consistent with nocturnal resource and fatigue effects (40). Key implications to reduce such risk include implementing nighttime delirium bundles to reduce triggers and manage “sundowning” (41); strengthening night staffing and escalation pathways given higher violence risk on these shifts (34); and using agitation/delirium protocols rather than relying on melatonin or ramelteon (42), which show only uncertain benefit in trials.

A Chinese survey including 306 ICU nurses revealed that 78% had experienced violence, with associations to burnout and turnover intention, partly buffered by organizational commitment (32). This has consequences with up to 22% of nurses reported leaving their workplace due to violence and 13% suffering anxiety, sleep problems, and stress (15).

Patient consequences, less studied, were quantified in our study with 60% of incidents causing complications, mainly the necessity of increasing sedation and restraint-related harms. This aligns with an American cross-sectional analysis, which showed nurse exposure to violence being correlated with lower patient safety climate scores (43).

Despite these insights, interventions remain weak (15). These included education sessions, a program on WPV definitions, crisis prevention, and online reporting, which increased staff confidence but not events with a stronger safety culture linked to lower odds of WPV. By contrast, the identification of verbal violence and aggression at index being associated with recurrence in our present study provides a practical, ICU-specific cue, which could be further enhanced using artificial intelligence (44).

This study represents one of the largest systematic investigations of aggression and violence directed against ICU nurses in a European tertiary care setting. The structured integration of incident reporting into daily ICU workflow ensured consistent and contemporaneous data capture, minimizing the risk of missing events. The comprehensive dataset, spanning both patient- and nurse-related characteristics as well as subsequent interventions, allowed for a perspective on the epidemiology, risk factors, and outcomes of aggressive and violent incidents. The use of robust statistical methods enhances the validity of the findings.

The retrospective single-center nature of the study limits the generalizability to other ICUs. Furthermore, the retrospective approach risks recall bias, as nurses may have recalled and documented incidents differently if there was a delay between the event and the report. The reliance on the categorization of incidents as aggression vs. WPV upon the caregivers’ free-text and discretion represents another limitation. Nevertheless, embedding documentation into routine shift reporting likely reduced this risk, and the high completeness of reporting supports the reliability of the data. Due to the risk of denominator bias from anonymous admissions and extreme variations in patient “time-at-risk,” we could not calculate a formal incidence rate. However, highlighting that 14% of all examined shifts involved reports of aggression or violence is perhaps an even more critical and informative finding, as it directly illustrates the high frequency with which ICU nurses must contend with such incidents in their daily practice. Anonymous data collection prevented the identification and thereby the comparison of reporting vs. nonreporting nurses. Furthermore, comparing patients involved in incidents to the general ICU population was beyond the study’s scope. Additionally, analyses of recurrence should be interpreted as exploratory and hypothesis-generating rather than establishing causal relationships.

## CONCLUSIONS

Violence in the ICU is frequent and often caused by patients. Recurring violence was associated with longer ICU stay, initial aggression, and verbal violence. Staff harm was rare, but incidents were associated with complications in patients. Validating these associations is essential for violence forecasting, alongside implementing ICU-specific de-escalation and support to enhance staff safety.

## ACKNOWLEDGMENTS

We thank Rita Achermann for performing the data queries and for her substantial contributions to the development of the R code used for the screening.

## Supplementary Material
