# Supplementary material for: Critical Care Aggression and Violence Events (CAVE) Against Nurses in the ICU: Epidemiology and Risk Factors for Recurrence
Source: Crit Care Med. 2026 Jul 24;54(10):2728–40. doi: 10.1097/CCM.0000000000007264 (PMC13632810; doi:10.1097/CCM.0000000000007264)
Supplement: Supplementary file 1 [file ccm-54-2728-s001.pdf]

Supplemental Tables

Supplemental Table 1: Keywords used for screening workplace violence in the ICU using regular expression patterns

Category	Keywords*
General	assault; rejecting; defensive; agitated; security staff; Pantex (private security company); security service; police; danger; violence; aggressive; uncooperative; abusive; harassment; abuse
Physical	physical assault; hitting; punching; scratching; spitting; pinching; damaging; attacking; assaulting; endangering; slapping; strangling; throwing; injuring; terror; restrictive measures; coercive measures
Verbal	insulting; bullying; humiliating; shouting; yelling; swearing; loud; cursing; irritable; threatening; blackmailing; demanding; confrontational; hostile; manipulative
Non-verbal	middle finger; obscene gesture; clenched fists; threatening gestures; intimidating body language
Sexual	obscene; intrusive; sexual; kissing; touching; harassing; offensive; suggestive; pornographic; dirty; indecent; lewd; ambiguous

*Translated from the original German terms.

Supplemental Table 2: Baseline characteristics of involved individuals with aggression and/or violence in the ICU

**Characteristics of involved individuals (n=308)**	**n (%) or median (IQR)**
Involved individuals, n (%)	
Patient	300 (97.4)
Relative	8 (2.6)
Age in years, median (IQR)	66.5 (49.0–77.5)
Male sex, n (%)	220 (71.4%)
ICU LOS in days, median (IQR)	4.4 (1.9– 9.4)
Origin of admission, n (%)	
Emergency department	134 (43.5)
General wards	95 (30.8)
Other hospital, emergency department	23 (7.8)
Other hospital, general wards	15 (4.9)
Other hospital, ICU	9 (2.9)
Intermediate care unit	27 (8.8)
Psychiatric clinic	4 (1.3)
Postoperative admission, n (%)	78 (25.3)
Emergency admission, n (%)	276 (89.6)
Ventilator support at admission, n (%)	250 (81.2)
Invasive mechanical ventilation	100 (40.0)
BIPAP	7 (2.8)
HFNC	18 (7.2)
Oxygen support	125 (50.0)
Vasopressor support at admission, n (%)	116 (37.7)
Charlson Comorbidity Index, median (IQR)	4 (2-6)
SAPS 2, median (IQR)	36 (27-44)
GCS at admission, median (IQR)	11 (3-14)
RASS at admission, median (IQR)	-1 (-4-1)
ICDSC at admission, median (IQR)	3 (0-5)
Prior ICU stays, n (%)	123 (39.9)
Number of prior stays, median (IQR)	1.0 (1.0– 3.0)
Aggression and/or violence documented prior to ICU stay, n (%)	52 (16.9)
Advanced directives available, n (%)	45 (14.6)
Legal guardian appointed, n (%)	24 (7.8)
Marital Status, n (%)	
Single	90 (29.2)
Relationship	24 (7.8)
Married	136 (44.2)
Divorced	15 (4.9)
Widowed	20 (6.5)
Not documented	25 (8.1)
Living situation, n (%)	
with family	164 (53.2)
alone	96 (31.2)
assisted living	16 (5.2)
nursing home	13 (4.2)
homeless	14 (4.5)
asylum center	5 (1.6)
Swiss nationality, n (%)	226 (73.4)
German language, n (%)	223 (72.4)
Main Diagnosis, n (%)	
Neurologic disease	88 (28.6)
Psychiatric disease	19 (6.2)
Metabolic disease	40 (13)
Infectious disease	37 (12)

Cardiovascular disease	55 (17.9)
Gastrointestinal disease	14 (4.5)
Pulmonary disease	29 (9.4)
Trauma	17 (5.5)
Other	11 (3.6)
Type of neurologic disease, n (%)	88 (28.6)
Delirium, n (%) [% of 88]	28 (9.0) [31.8]
Epilepsy, n (%) [% of 88]	18 (5.8) [20.4]
Stroke, n (%) [% of 88]	13 (4.2) [14.8]
Traumatic brain injury, n (%) [% of 88]	11 (3.6) [12.5]
Encephalitis, n (%) [% of 88]	5 (1.6) [5.7]
CNS infection, n (%) [% of 88]	1 (0.3) [1.1]
CNS surgery, n (%) [% of 88]	1 (0.3) [1.1]
Brain tumor, n (%) [% of 88]	1 (0.3) [1.1]
Other, n (%) [% of 88]	10 (3.25) [1.4]
Psychiatric disease, n (%)	19 (6.2)
Dissociative disease, n (%) [% of 19]	5 (1.6) [26.3]
Psychosis, n (%) [% of 19]	4 (1.3) [21.1]
Other, n (%) [% of 19]	13 (4.2) [68.4]
Metabolic disease, n (%)	40 (13.0)
Intoxication with medication, n (%) [% of 40]	13 (4.2) [32.5]
Drug intoxication, n (%) [% of 40]	9 (2.9) [22.5]
Dselectrolytemia, n (%) [% of 40]	6 (2) [15.0]
Multiple organ failure, n (%) [% of 40]	3 (1.0) [7.5]
Alcohol intoxication, n (%) [% of 40]	2 (0.7) [5.0]
Hepatic encephalopathy, n (%) [% of 40]	2 (0.7) [5.0]
Uremia, n (%) [% of 40]	1 (0.3) [2.5]
Diabetic ketoacidosis, n (%) [% of 40]	1 (0.3) [2.5]
Hyperglycemia, n (%) [% of 40]	1 (0.3) [2.5]
Hypoglycemia, n (%) [% of 40]	1 (0.3) [2.5]
Infectious disease, n (%)	37 (12.0)
Sepsis, n (%) [% of 37]	22 (7.1) [59.5]
Septic shock, n (%) [% of 37]	12 (3.9) [32.4]
Endocarditis, n (%) [% of 37]	2 (0.7) [5.4]
Pneumonia, n (%) [% of 37]	1 (0.3) [2.7]
Positive toxicologic screen, n (%)	43 (14.0)
Benzodiazepines, n (%) [% of 43]	25 (8.1) [58.1]
Cocaine, n (%) [% of 43]	19 (6.1) [44.2]
THC, n (%) [% of 43]	17 (5.5) [39.5]
Opiates, n (%) [% of 43]	5 (1.6) [11.6]
TCA/Antidepressants, n (%) [% of 43]	3 (0.9) [0.7]
Other, n (%) [% of 43]	16 (5.2) [37.2]
History of neurologic disease, n (%)	109 (35.3)
Epilepsy (n, %, [% of 109]	41 (13.3) [37.6]
Stroke, n (%) [% of 109]	29 (9.4) [26.6]
Delirium, n (%) [% of 109]	25 (8.1) [22.9]
Other, n (%) [% of 109]	17 (5.5) [15.6]
Traumatic brain injury, n (%) [% of 109]	13 (4.2) [11.9]
Brain hemorrhage, n (%) [% of 109]	12 (3.9) [11.0]
CNS surgery, n (%) [% of 109]	7 (2.3) [6.4]
Encephalitis, n (%) [% of 109]	2 (0.6) [1.8]
CNS infection, n (%) [% of 109]	1 (0.3) [0.9]
History of psychiatric disease, n (%)	88 (28.6)
Depression, n (%) [% of 88]	27 (8.8) [30.7]
Borderline personality disorder, n (%) [of 88]	26 (8.4) [29.5]
PTSD, n (%) [% of 88]	15 (4.9) [17.0]
Psychosis, n (%) [% of 88]	11 (3.6) [12.5]
Dissociative disorder, n (%) [% of 88]	11 (3.6) [12.5]
Bipolar disorder, n (%) [% of 88]	6 (1.9) [6.8]
Schizophrenia, n (%) [% of 88]	4 (1.3) [4.5]

Narcistic personality disorder, n (%) [% of 88]	4 (1.3) [4.5]
Histrionic personality disorder, n (%) [% of 88]	3 (1.0) [3.4]
Other, n (%) [% of 88]	33 (10.7) [30.6]
History of alcohol abuse, n (%)	106 (34.4)
History of drug abuse, n (%)	61 (19.8)
History of dementia, n (%)	35 (11.4)

IQR = Interquartile range; BMI = body mass index; ICU = intensive care unit; LOS = length of stay; CPR = cardiopulmonary resuscitation; CNS = central nervous system; PTSD = post-traumatic stress disorder; THC = tetrahydrocannabinol; TCA = tricyclic antidepressants; GCS = Glasgow Coma Score (ranging from 3 to 15); RASS = Richmond agitation-sedation score; ICDSC = intensive care delirium screening checklist; BIPAP = bilevel positive airway pressure; HFNC = high-flow nasal cannula; SAPS 2 = simplified acute physiology score 2 (ranging from 0 to 163)

Supplemental Table 3: Baseline characteristics of victims among ICU nurses

Victim characteristics (n=308)	n (%) or median (IQR)
Job title, n (%)	
ICU nurse<br>Registered nurse	273 (88.6)<br>35 (11.4)
Age in years, median (IQR)	36 (29-46)
Female sex, n (%)	204 (66.2)
Swiss nationality, n (%)	99 (32.1)
Working percentage, median (IQR)	90 (70-100)
Working experience in years, median (IQR)	7.4 (3.1-11.3)
Additional violent incidents by the same patient, n (%)	43 (14.0)
New (i.e., first time) victim of violent behavior, n (%)	39 (12.7)

IQR = Interquartile range; ICU = intensive care unit; GCS = Glasgow Coma Score (ranging from 3 to 15); RASS = Richmond agitation-sedation score

Supplemental Table 4: Univariable comparisons between patients being aggressive (without violent behavior) and patients that become violent after initially being aggressive

Characteristics	Only aggressive (n=76)	Violent after aggression (n=50)	p-value*
**Perpetuator characteristics**			
Age in years, median (IQR)	69.5 (52-78)	62.5 (42-72)	0.14[1]
Male sex, n (%)	51 (67.1)	40 (80.0)	0.11[2]
ICU LOS, median (IQR)	4 (2-9.8)	5.8 (3.3-14.6)	0.01[1]
Prior ICU stays, n (%)	34 (44.7)	15 (30.0)	0.10[2]
Postoperative admission, n (%)	14 (18.4)	15 (30.0)	0.13[2]
Emergency admission, n (%)	69 (90.8)	48 (96.0)	0.31[3]
Advanced directives available, n (%)	13 (17.1)	5 (10.0)	0.27[2]
Legal guardian appointed, n (%)	6 (7.9)	3 (6.0)	1.00[3]
Marital status, n (%)			0.85[3]
Single	23 (30.3)	19 (38.0)	
Relationship	5 (6.6)	5 (10.0)	
Married	37 (48.7)	18 (36.0)	
Divorced	3 (3.9)	2 (4.0)	
Widowed	3 (3.9)	2 (4.0)	
Not documented	6 (7.9)	4 (8.0)	
Living situation, n (%)			0.44[3]
with family	45 (59.2)	29 (58.0)	
alone	20 (26.3)	13 (26.0)	
assisted living	3 (3.9)	4 (8.0)	
nursing home	4 (5.2)	1 (2.0)	
homeless	4 (5.2)	1 (2.0)	
asylum center	0 (0.0)	2 (4.0)	
Swiss nationality, n (%)	60 (78.9)	31 (62.0)	0.04[2]
German language (n, %)	61 (80.3)	28 (56.0)	0.003[2]
Main diagnosis, n (%)			0.10[3]
Neurologic disease	21 (27.6)	12 (24.0)	
Psychiatric disease	4 (5.3)	5 (10.0)	
Metabolic disease	10 (13.2)	7 (14.0)	
Infectious disease	10 (13.2)	3 (6.0)	
Cardiovascular disease	11 (14.5)	9 (18.0)	
Gastrointestinal disease	2 (2.6)	2 (4.0)	
Pulmonary disease	12 (15.8)	3 (6.0)	
Trauma	1 (1.3)	7 (14.0)	
Positive toxicologic screening, n (%)	14 (18.4)	11 (22.0)	0.62[2]
Charlson comorbidity index, median (IQR)	4 (1-6)	3 (0-6)	0.36[1]
SAPS 2, median (IQR)	35 (26.5-45)	36 (27-42)	0.99[1]
GCS at incident, median (IQR)	13 (12-14)	13 (12-14)	0.10[1]
RASS at incident, median (IQR)	3 (2-4)	4 (3-4)	**0.001[1]**
ICDSC at incident, median (IQR)	4.5 (2-6)	6 (5-7)	**<0.001**
Ventilator support at admission, n (%)	54 (71.1)	41 (82.0)	0.16[2]
Vasopressor support at admission, n (%)	25 (32.9)	20 (40.0)	0.42[2]
History of neurologic disease, n (%)	76 (33.8)	33 (39.8)	0.33[2]
History of psychiatric disease, n (%)	63 (28.0)	25 (30.1)	0.72[2]
History of alcohol abuse, n (%)	21 (27.6)	20 (4.0)	0.15[2]
History of drug abuse, n (%)	16(21.1)	15 (30.0)	0.25[2]
History of dementia, n (%)	11 (14.5)	2 (4.0)	0.06[2]
Violence prior to ICU stay, n (%)	8 (10.5)	9 (18.0)	0.23[2]
**Victim characteristics**			
ICU nurse (n, %)	67 (88.2)	45 (90.0)	0.75[2]
Registered nurse (n, %)	9 (11.8)	5 (10.0)	0.75[2]

Female sex (n, %)	51 (67.1)	30 (60.0)	0.42
Age in years, median (IQR)	40 (30.5-45.5)	35 (29-49)	0.86[1]
Working percentage, median (IQR)	90 (70-100)	90 (80-100)	0.44[1]
Working experience in years, median (IQR)	8.1 (3.2-10.1)	8.2 (3.7-11.4)	0.70[1]
Swiss nationality, n (%)	20 (26.3)	18 (36.0)	0.25[2]
**Clinical characteristics**			
Time from admission to incident in days, median (IQR)	1.2 (0.3-4.3)	3.2 (1.2-7.9)	**0.001[1]**
Type of shift during incident, n (%)			0.91[2]
Day	26 (34.2)	16 (32.0)	
Late	26 (34.2)	19 (38.0)	
Night	24 (31.6)	15 (30.0)	
Time since last shift in hours, median (IQR)	3 (1.6-5.5)	3.5 (2-6)	0.53[1]
Restrained at first incident, n (%)	16 (21.0)	24 (48.0)	**0.001[2]**
Measures taken, n (%)			
Called other nurses	3 (3.9)	5 (10.0)	0.17[2]
Called doctor	7 (9.2)	9 (18.0)	0.15[2]
Called security	3 (3.9)	4 (8.0)	0.43[3]
Verbal soothing	55 (72.4)	33 (66.0)	0.45[2]
Involved family	4 (5.2)	8 (16.0)	0.045[2]
Administered sedatives	34 (44.7)	38 (76.0)	**0.001[2]**
Administered antipsychotics	24 (31.6)	24 (48.0)	0.06[2]
Applied restraints	15 (19.7)	23 (46.0)	0.002[2]
Offered distraction (TV, music)	2 (2.6)	6 (12.0)	0.04[2]
One on one nursing	17 (22.4)	16 (32.0)	0.23[2]
Patient's complications, n (%)			
Escalation of sedation	26 (34.2)	34 (68.0)	**<0.001[2]**
More tubes/lines/catheters	0 (0.0)	2 (4.0)	0.16[3]
Less tubes/lines/catheters	2 (2.6)	2 (4.0)	0.65[3]
Physical injuries	0 (0.0)	1 (2.0)	0.40[3]
Emotional injuries	0 (0.0)	3 (6.0)	0.06[3]
Aspiration	0 (0.0)	1 (2.0)	0.40[3]
Pain	0 (0.0)	1 (4.0)	0.16[3]
Agitation	2 (2.6)	3 (6.0)	0.39[3]

[1] Mann-Whitney-U; [2] Pearson Chi-Square; [3] Fisher's exact
ICU = intensive care unit; LOS = length of stay; GCS = Glasgow Coma Score (ranging from 3 to 15); RASS = Richmond agitation-sedation score; ICDSC = intensive care delirium screening checklist; BIPAP = bilevel positive airway pressure; SAPS 2 = simplified acute physiology score 2 (ranging from 0 to 163)
*Level of significance adjusted using the Bonferroni correction for multiple comparisons and set at a significance level at a two-sided p-value of ≤0.001 due to the Bonferroni correction
Bold font indicating significance

Supplemental Table 5: Uni- and multivariable analyses regarding characteristics associated with ICU patients transforming from aggression to violent behavior (n=126)

**Characteristics associated with patients' repetitively aggressive and/or violent**	**Univariable analyses**			**Multivariable analyses**		
	**uaOR**	**95% CI**	**p-value**	**aOR**	**95% CI**	**p-value***
RASS at incident	2.10	1.41-3.14	**<0.001**	1.72	1.06-2.77	0.027
ICDSC at incident	1.41	1.16-1.71	**0.001**	1.27	1.03-1.57	0.025
Restrained at first incident	3.46	1.58-7.57	0.002	2.95	1.19-7.28	0.019
Time from admission to incident	1.00	1.00-1.01	0.034	1.00	1.00-1.01	0.035
Sedatives administered following first incident	3.91	1.77-8.63	**0.001**	1.88	0.55-6.36	0.312
Escalation of sedation following first incident	4.09	1.91-8.74	**<0.001**	1.59	0.51-495	0.424

uaOR = unadjusted odds ratio; aOR = adjusted odds ratio; CI = confidence interval; RASS = Richmond agitation-sedation score; ICDSC = intensive care delirium screening checklist
*Level of significance adjusted using the Bonferroni correction for multiple comparisons and set at a significance level at a two-sided p-value of ≤0.001 due to the Bonferroni correction
Hosmer Lemeshow goodness-of-fit test Chi^2 =3.58; p=0.893 indicating adequate model fit
Bold font indicating significance
